# Symbiotic Effectiveness of Rhizobial Mutualists Varies in Interactions with Native Australian Legume Genera

**DOI:** 10.1371/journal.pone.0023545

**Published:** 2011-08-26

**Authors:** Peter H. Thrall, Anna-Liisa Laine, Linda M. Broadhurst, David J. Bagnall, John Brockwell

**Affiliations:** 1 Commonwealth Scientific and Industrial Research Organisation (CSIRO) Plant Industry, Canberra, ACT, Australia; 2 Metapopulation Research Group, Department of Biosciences, University of Helsinki, Helsinki, Finland; Freie Universität Berlin, Germany

## Abstract

**Background and Objectives:**

Interactions between plants and beneficial soil organisms (e.g. rhizobial bacteria, mycorrhizal fungi) are models for investigating the ecological impacts of such associations in plant communities, and the evolution and maintenance of variation in mutualisms (e.g. host specificity and the level of benefits provided). With relatively few exceptions, variation in symbiotic effectiveness across wild host species is largely unexplored.

**Methods:**

We evaluated these associations using representatives of several legume genera which commonly co-occur in natural ecosystems in south-eastern Australia and an extensive set of rhizobial strains isolated from these hosts. These strains had been previously assigned to specific phylotypes on the basis of molecular analyses. In the first of two inoculation experiments, the growth responses of each host species was evaluated with rhizobial strains isolated from that species. The second experiment assessed performance across genera and the extent of host specificity using a subset of these strains.

**Results:**

While host growth responses to their own (sympatric) isolates varied considerably, rhizobial phylotype was a significant predictor of symbiotic performance, indicating that bacterial species designations on the basis of molecular markers have ecological importance. Hosts responded in qualitatively different ways to sympatric and allopatric strains of rhizobia, ranging from species with a clear preference for their own strains, to those that were broad generalists, through to species that grew significantly better with allopatric strains.

**Conclusion:**

Theory has focused on trade-offs between the provision of benefits and symbiont competitive ability that might explain the persistence of less beneficial strains. However, differences in performance among co-occurring host species could also drive such patterns. Our results thus highlight the likely importance of plant community structure in maintaining variation in symbiotic effectiveness.

## Introduction

Interactions between plants and symbiotic soil microbes are major determinants of ecosystem productivity and diversity. Plants can receive substantial benefits from root associated symbionts, such as rhizobial bacteria and mycorrhizal fungi, particularly where increased nutrient availability can provide hosts with significant fitness advantages. The presence of effective mutualists enhances the growth and competitive ability of host plants, and in turn can influence successional dynamics [1], plant productivity [2], and community restoration [3]–[5]. Plant-associated microbes, including pathogens, are also important regulators of plant community dynamics and structure [6], [7]. The general importance of plant-soil microbe interactions for community assembly and coevolutionary processes, and the value of utilising these associations in the management and restoration of functioning native ecosystems [4], [5], [8] is widely recognised. However, considerable empirical and theoretical gaps remain in our ecological and evolutionary understanding of plant-soil symbiont interactions in diverse natural host communities.

Characterisation of the degree to which symbiotic microbes vary in the provision of mutualistic benefits in relation to environmental quality, host species and plant community structure is critical to developing an understanding of their role as agents of productivity and selection in natural populations [9]. It is becoming increasingly clear that plants and microbes interact within a diverse community of potential partners and competitors [10], within which interactions vary widely in both specificity (i.e. host range) and position along the mutualism-parasitism continuum. Furthermore, variation in host and microbe genetic identity can strongly influence the strength, net fitness effect and even direction of symbiotic interactions [11], as has been shown for *Acacia* spp. and associated rhizobia [12]–[15]. Much less information is available regarding interactions across host genera, although some previous work indicates that patterns of host specificity and symbiotic effectiveness are likely to be complex [16], [17].

The persistence of ineffective symbionts (‘cheaters’) in mutualistic associations has been a focus of evolutionary theory as well as empirical studies, with a particular emphasis on legume-rhizobial interactions [18]–[21]. For some mutualists such as mycorrhizal fungi, there is evidence for trade-offs between growth promotion and inter-strain competitiveness (i.e. a cost of mutualism) which could mediate ‘main-effect’ differences among fungal strains [22], [23]. Such trade-offs are also likely to play a role in maintaining variation in legume-rhizobial interactions [19]. However, given the potential for considerable host specificity in such interactions, whether particular rhizobial strains are characterised as ineffective is likely to be at least partly context dependent (i.e. strains perceived as essentially parasitic on one host may well be beneficial on another host), as has been shown for mycorrhizal fungi [24]. Thus, likely determinants of the extent of variation in mutualistic benefits within and among host species include spatial structure in host distribution and community structure, and environmental quality [9], [25]. Overall, characterisation of these associations in nature is still insufficient to determine the extent to which outcomes are determined by variation among rhizobial strains, host species which may differ in their ability to discriminate among strains, or by their interaction, although clearly genotype×genotype interactions are an important determinant of the level of mutualistic benefits conferred [11].

Development of a more quantitative understanding of the genetic, ecological and environmental factors that drive the evolution of host range and mutualistic benefit also demands characterisation of the correspondence between phylotypes identified using molecular approaches and their ecological performance. For example, in soil microbial ecology, molecular studies have revealed a myriad of previously cryptic bacterial species [26]–[28] which could represent functionally meaningful diversity should genetically distinct taxa also have correspondingly different physiologies and ecologies. However, examples confirming distinct ecologies of phylogenetically distinct taxa (phylotypes) within a lineage are limited [29]–[31]. Thus, the generality of the correspondence between genetic delineation of microbial species and ecological function is uncertain [32]–[34], although a recent study [35] provides strong support for such a correspondence in native legume-rhizobial associations. This is problematic because the link between genes and ecology is a basic assumption underlying the rapidly growing fields of molecular ecology and environmental microbiology [36].

In the past decade, several studies have examined the phenotypic and genetic diversity, and phylogenetic relationships of rhizobia in native Australian associations [37]–[41]. *Bradyrhizobium* is the most common genus of root-nodule bacteria reported as nodulating Australian native legumes [37], but many other nodule-forming bacterial genera are recorded on leguminous genera represented by Australian natives, including *Rhizobium*, *Mesorhizobium*, *Sinorhizobium* (*Ensifer*), *Burkholderia*, *Devosia*, *Phyllobacterium* [14], [15], [37], [41] and *Ochrobactrum* [42]. To what extent these genera are involved in cross nodulation and meaningful symbiotic relationships in the field with Australian native legumes is unknown. However, recent work suggests that at least some *Acacia* spp. form significant associations with many of these bacterial genera [41], [43], and that these generic associations may vary both in relation to environmental factors such as soil salinity [13], [14] and geographic distance [43], [44].

Here we use native representatives of several host genera in the Fabaceae, and an extensive collection of rhizobial strains from these hosts to: a) characterise variation within host genera in symbiotic effectiveness; b) evaluate how these patterns change across co-occurring host genera, both in terms of average effectiveness and host specificity; and c) evaluate the extent to which rhizobial phylotypes (i.e. designated on the basis of molecular markers) have ecological significance with regard to predicting plant growth responses. As noted above, in addition to the potential of such studies to contribute to basic understanding of host-symbiont associations, there is considerable applied value in understanding these relationships better with regard to improving the potential to re-establish functional and diverse native plant communities [4], [5], [45].

## Materials and Methods

In this study, we examined the growth performance of eight native legume species from different genera (all in the Fabaceae) in replicated glasshouse inoculation trials using a broad range of rhizobial strains. The host species were: *Bossiaea foliosa* A. Cunn., *Daviesia ulicifolia* C.R.P. Andrews, *Dillwynia retorta* (Wendl.) Druce, *Goodia lotifolia* Salisb., *Hardenbergia violacea* (Schneev.) Stearn, *Indigofera australis* Willd., *Oxylobium ellipticum* (Vent.) R. Br. and *Podolobium ilicifolium* (Andrews) Crisp and P.H. Weston. All eight species are common inhabitants of infertile soils in south-eastern Australia.

Symbiotic relationships were evaluated in two experiments in which we measured the extent of nodulation and plant growth responses. In the first experiment, we inoculated each plant host species with rhizobial strains isolated from nodules of that species growing naturally in the field. In the second experiment, we cross-inoculated a subset of these host species with rhizobial strains from the other hosts, as well as with its own strains.

In addition to the field isolates obtained from the target host genera, an additional strain (2836) was used in the first glasshouse experiment. This strain was from the collection of Lafay & Burdon [37] and it has previously been shown experimentally to fix nitrogen effectively with a diverse range of Australian native species of *Acacia*. Strain 2836 was originally isolated from *Acacia melanoxylon* R. Br. and is a component of “Wattlegrow” (a commercial inoculant for native Australian legumes; Becker Underwood, Somersby, New South Wales). In the results that follow, control strain 2836 has been designated as “WG”.

### Rhizobial Strains

#### Isolation of rhizobial strains from native hosts

Nine general localities in south-eastern Australia were chosen (Table 1), with one or more of the eight legume hosts used in this study), occurring at each site [37]. Vigorous adult plants were dug up in order to isolate rhizobial bacteria from their root nodules. Isolates of root-nodule bacteria (Table 1) were extracted from nodules using standard techniques [46]. Pure cultures of 130 isolates were suspended in yeast mannitol broth [47] and stored under glycerol at −80°C.

**Table 1:** Location of sampling sites and host of origin for strains of root-nodule bacteria isolated from the nodules of 8 Australian native legumes used in the glasshouse inoculation trials [see 37].

Sampling Site (coordinates)	Host Species (tribe)	Phylotype: rhizobial strains
Lob's Hole, NSW (35°39′S, 148°25′E)	*Bossiaea foliosa* (Bossiaeae)	**A:** 6046; **B:** 5049, 5050, 5053, 5058, 5060, 5061; **Q:** 5052
Mt Franklin Rd, ACT (35°19′S, 148°50′E)	*B. foliosa*	**A:** 5069, 5070; **P:** 5064
Island Bend, NSW (36°19′S, 148°29′E)	*B. foliosa*	**A:** 6058; **F:** 5913, 5914, 5916; **I:** 6059; **S:** 5911
	*Daviesia ulicifolia* (Mirbelieae)	**I:** 5925, 5929
Two Sticks Rd, NSW (35°16′S, 148°51′E)	*D. ulicifolia*	**A:** 5147**+**; **Q:** 5140, 5143, 5146, 5153
	*Oxylobium ellipticum (Mirbelieae)*	**A:** 5514, 5517, 5790; **O:** 5804
Lowden Forest Park Rd, NSW (35°31′S, 149°34′E)	*D. ulicifolia*	**A:** 5159, 5166, 5177**+**; **D:** 5863; **F:** 5169; **H:** 5170; **O:** 5185; **P:** 5174**+**, 5175
	*Goodia lotifolia* (Bossiaeae)	**A:** 5331, 5345, 5386, 5765; **F:** 5363, 5368, 5372**+**, 5395; **H:** 5354**+**, 5375**+**, 5393; **I:** 5387, 5871; **M:** 5365; **P:** 5332, 5359, 5390
	*Podolobium ilicifolium* (Mirbelieae)	**A:** 5550; **E:** 5810; **P:** 5548, 5552, 5812
Black Mountain, ACT (35°16′S, 149°06′E)	*Dillwynia retorta* (Mirbelieae)	**A:** 5282, 5284, 5286, 5287, 5290**+**, 5292, 5293, 5294, 5295, 5296, 5298, 5300, 5301, 5302, 5304**+**, 5306+, 5307
	*Hardenbergia violacea* (Phaseoleae)	**A:** 5412, 5414**+**, 5417, 5418, 5422, 5892; **L:** 5413**+**; **Q:** 5410, 5411, 5415, 5421
Turpentine Road, NSW (35°02′S, 150°26′E)	*D. ulicifolia*	**A:** 5195
	*P. ilicifolium*	**A:** 5555, 5556, 5558; **B:** 5557, 5561
Gunning Road, NSW (34°47′S, 149°16′E)	*H. violacea*	**A:** 5401, 5403, 5407, 5408, 5865**+**; **B:** 5409
Boboyan Road, ACT (35°52′S, 148°56′E)	*Indigofera australis* (Indigoferae)	**A:** 5452, 5453**+**, 5454, 5455**+**, 5458, 5460, 5461, 5463, 5466, 5479, 5781, 5857**+**; **Q:** 5457
	*O. ellipticum*	**A:** 5519, 5520, 5522, 5523, 5524, 5525, 5526, 5527, 5532, 5533, 5535; **P:** 5529, 5537
Ben Boyd National Park, NSW (37°13′S, 150°49′E)	*P. ilicifolium*	**A:** 5562**+**, 5564, 5566**+**; **D:** 5563; **P:** 5565**+**

All strains are *Bradyrhizobium* spp., except those designated as phylotype Q which are *Rhizobium* spp. Those strains marked with ‘+’ represent the subset used in Expt II that were characterised as symbiotically effective on their own host species in Expt I.

#### Classification of rhizobial phylotypes and generic affiliation

The 130 field isolates from the nine sites (Table 1) and the *Bradyrhizobium* control strain (WG) were genetically characterised and assigned at the generic level by Lafay & Burdon [37] as part of a larger research effort investigating rhizobial diversity on native legumes in southeastern Australia. In that study, field-collected strains were assigned unique phylotype profiles on the basis of RFLP banding patterns from multiple enzymes. Generic affiliations of representative isolates from each phylotype were determined following phylogenetic analyses of SSU rDNA sequences. The majority of these strains were *Bradyrhizobium* spp (representing 12 different phylotypes) with ten *Rhizobium* strains (representing a single phylotype) isolated from *B. foliosa*, *Daviesia ulicifolia*, *H. violacea* and *I. australis*. Summary information on host species, collection sites and generic affiliation of the rhizobial strains used in the present study are given in Table 1; further details of the distribution of phylotypes by host species and geographic location are given in Lafay & Burdon [37].

### Glasshouse Inoculation Studies

For the glasshouse inoculation studies of symbiotic effectiveness and host specificity, seed of each legume species was obtained from the Australian Seed Company, Hazelbrook, New South Wales, or from the CSIRO Australian Tree Seed Centre, Canberra, ACT. In the first glasshouse experiment evaluating within-host performance (Experiment I), seedlings of all 8 host genera were separately inoculated with rhizobial strains that had been isolated from nodules of that host. For each host species, a total of 17 strains were used as individual inocula (Table 1), with the exception of *P. ilicifolium* (15 strains) and *I. australis* (13 strains). As noted above, each host species was also separately inoculated with acacia strain WG. Two uninoculated control treatments were also included where plants either received a full nutrient solution including nitrogen or a nutrient solution lacking nitrogen (designated as N^+^ and N^−^ and respectively). All inoculated plants received the N^−^ nutrient solution.

The second glasshouse experiment was designed to evaluate symbiotic benefits across host genera (Experiment II). The three most effective strains from each host species (except for *B. foliosa* and *O. ellipticum* which showed a general lack of responsiveness to rhizobial inoculation in the first trial) were selected on the basis of their N_2_-fixing performance in Experiment I. These strains were used as inocula in Experiment II (Table 1). In this trial, each host was inoculated with its own three strains (referred to as ‘sympatric’) as well as those from each of the other genera (i.e. ‘allopatric’). As in Experiment I, uninoculated control treatments (N^−^, N^+^) were also included.

For both experiments, seeds were pretreated with boiling water for 1 minute, allowed to cool and imbibe for 24 hrs, surface sterilized with ethanol (98%) for 30 seconds then with sodium hypochlorite (5%) for 10 minutes, rinsed 10 times with sterile distilled water, sown into a shallow dish of sterile, moist horticultural vermiculite, and incubated at 25°C until emergence. Newly-emerged seedlings were transplanted (1 per pot) into cylindrical (8 cm×15 cm) polyethylene pots containing a mixed substrate (1∶1 by volume) of steam-sterilised vermiculite and washed river sand. Seedlings were inoculated with a heavy suspension (approx. 1×10^9^ cells per plant) of monocultures of each rhizobial strain, or were left uninoculated as noted above. There were 10 replicates of each host×strain (or control) treatment.

Pots of each species were arranged in randomised blocks in a temperature-controlled glasshouse under standard day/night conditions (16 hrs at 25°C; 8 hrs at 18°C). Plants were watered daily with UV-sterilised tap water or as needed, and weekly with N-free McKnight's [48] solution. Plants in the N^+^ control group were given an additional 10 ml of H_2_0 containing 0.05% KNO_3_ once a week. Plants were harvested approximately 90 days after inoculation. At harvest, the roots were cut off and scored for occurrence and extent of nodule formation [43]: a) nodule number (0, <10, 10–50, >50), b) nodule functionality based on nodule colour and size [ranging from 1 (small non-N_2_-fixing nodules with white centres) to 5 (large N_2_-fixing nodules with pink/red centres), and c) nodule distribution [low scores (<2) represented plants with nodules distributed mostly in the root crown and higher scores (3–5) represented plants with nodules more broadly distributed throughout the root system]. All scoring was done by a single observer. Shoots were oven-dried (70°C for 48 hours) and weighed. Shoot dry weight was used as an index of rhizobial strain effectiveness at N_2_ fixation, given that this was the only source of N available for plant growth in the inoculation treatments.

### Statistical Analyses

There was a low level of nodulation of some uninoculated controls by rhizobial contaminants. In almost all instances where it occurred, contaminant nodulation did not lead to any appreciable N_2_ fixation as evidenced by the small plant dry weights in these treatments; therefore, it was ignored in data treatment. Plants in the N^+^ uninoculated control groups for *B. foliosa*, *D. ulicifolia* and *D. retorta* performed poorly in Experiment I, thus N^−^ controls were used as a more reliable benchmark of plant-to-plant variation in both glasshouse trials.

We measured the interaction between rhizobial strains and their host plants by calculating a response variable ‘symbiotic response’. For this purpose the dry weight of the host plants inoculated with different rhizobial strains was divided by the average dry weight of the uninoculated N^−^ control plants for that species. Hence, a value of one means that the host did not gain or lose anything from the rhizobial interaction relative to the N^−^ control treatment. Symbiotic response values <1 indicate that the rhizobial strain has a negative effect on its hosts compared to the null situation, and values >1 indicate a positive association between the host plant and a given rhizobial strain. For all host plant×rhizobia interactions we also examined nodule formation. This response variable (nodule presence/absence) has a binomial probability distribution and a logit link function. For all analyses, symbiotic response was confirmed to be normally distributed from the normal quantile plots following a log transformation. All analyses were done using SAS 9.1 [49]. Only significant interactions were included in the final models.

#### Experiment I: within-host variation

We first analyzed interactions between the different rhizobial isolates and their host plants using the entire dataset. We used a generalized linear model [50] for analysing whether isolates formed nodules with their hosts or not, and an ANOVA to analyze the symbiotic response (as described above). Host plant and isolate, nested under host plant, were the explanatory variables in the models. We then compared symbiotic response and nodule formation of strains originating from a given host to the generally effective strain WG on that same host as a ‘standardised’ measure of host response across the species used in our study. In this model WG *vs.* other strains was the fixed categorical explanatory variable nested under host plant. Strains, nested under host plant, were defined as random variables in the models.

We then analyzed whether phylotypes that were identified on the basis of earlier RFLP analyses [37] also represented biologically different functional units in their interaction with their host plant. We first asked whether rhizobial phylotypes colonizing the same host species differed in their interaction with that host. For this analysis we only included host genera (*Bossiaea*, *Daviesia*, *Goodia*, *Hardenbergia*, *Podolobium*) from which several rhizobial phylotypes had been identified. We used generalized linear mixed models (GLMMs) to analyze nodule formation and symbiotic response with host genus as a fixed factor in the model.

Phylotype, also a fixed factor, was nested within host genus, because different phylotypes were identified from different hosts. To control for variation among rhizobial strains, for both models we defined strains as random variables, nested under their phylotype and host genus, respectively. To test whether phylotypes performed differently across host species, we then analyzed a dataset of three host species (*B. foliosa*, *D. ulicifolia* and *G. lotifolia*) and four phylotypes that were isolated from each of them (A, F, I and P; Table 2). The models for analyzing patterns of nodule formation and symbiotic response were identical to the first phylotype analyses, except that here the effect of phylotype could be estimated across different host species and, hence, it was not nested in the analyses.

**Table 2:** Analyses of variation in nodulation and growth performance from the within-host inoculation trial (Experiment I) where each legume species was only inoculated with its own (sympatric) rhizobial strains.

Source	Estimate for random effects (± S.E.)	*Z/F*	*P* value
**Rhizobial isolates within host species**			
*Nodule formation*			
Host plant_7, 1101_		85.24	<0.0001
Isolate (Host plant)_122, 1101_		9.88	<0.0001
*Symbiotic response*			
Host plant_7, 1098_		140.89	<0.0001
Isolate (Host plant)_122, 1098_		7.63	<0.0001
**Phylotype: within host species**			
*Nodule formation*			
Strain (Phylotype, Host plant)	5.77±1.64	3.53	0.0002
Residual	0.47±0.02	19.25	<0.0001
Host plant_4, 53_		0.49	0.972
Phylotype (Host plant)_25, 53_		0.001	1.00
*Symbiotic response*			
Strain (Phylotype, Host plant)	0.69±0.15	4.59	<0.0001
Residual	0.81±0.04	19.09	<0.0001
Host plant_4, 53_		5.87	0.0005
Phylotype (Host plant)_25, 53_		2.32	0.0051
**Phylotype: across host species**			
*Nodule formation*			
Strain (Phylotype, Host plant)	2.36±1.03	2.30	0.0107
Residual	0.56±0.05	11.97	<0.0001
Host plant_2, 26_		4.52	0.0207
Phylotype_3, 26_		0.25	0.8608
*Symbiotic response*			
Strain (Phylotype, Host plant)	0.32±0.12	2.57	0.005
Residual	0.69±0.06	11.81	<0.0001
Host plant_2, 20_		22.71	<0.0001
Phylotype_3, 20_		4.42	0.0154
Host plant×Phylotype_6, 20_		4.08	0.0078

For the two phylotype level comparisons, analyses focused on subsets of hosts and rhizobial strains with sufficient representation (see Methods). *Z* statistics are given for random effects and *F* statistics for fixed effects.

#### Experiment II: among-genera interactions

The majority of the subset of rhizobial isolates chosen for the across-genera inoculation experiment were grouped within a single phylotype (Table 1). Analyses therefore focused on host origin of rhizobial strains as a predictor of plant growth. In particular, we tested whether host plant response to inoculation with their own sympatric rhizobial strains differed from their performance with strains isolated from other host species, measured as nodule formation and symbiotic response. In the first analysis the explanatory fixed variables in the GLMM models were host plant, the origin of the rhizobial strain, and their interaction. In the second model host plant and origin of the rhizobial strain were again designated as fixed explanatory variables, and we included a third explanatory variable for whether the interaction was sympatric or allopatric (as defined above). Rhizobial strain, nested within host origin, was defined as a random variable in all models.

## Results

### Experiment I: within-host species inoculations

**Figure 1:**
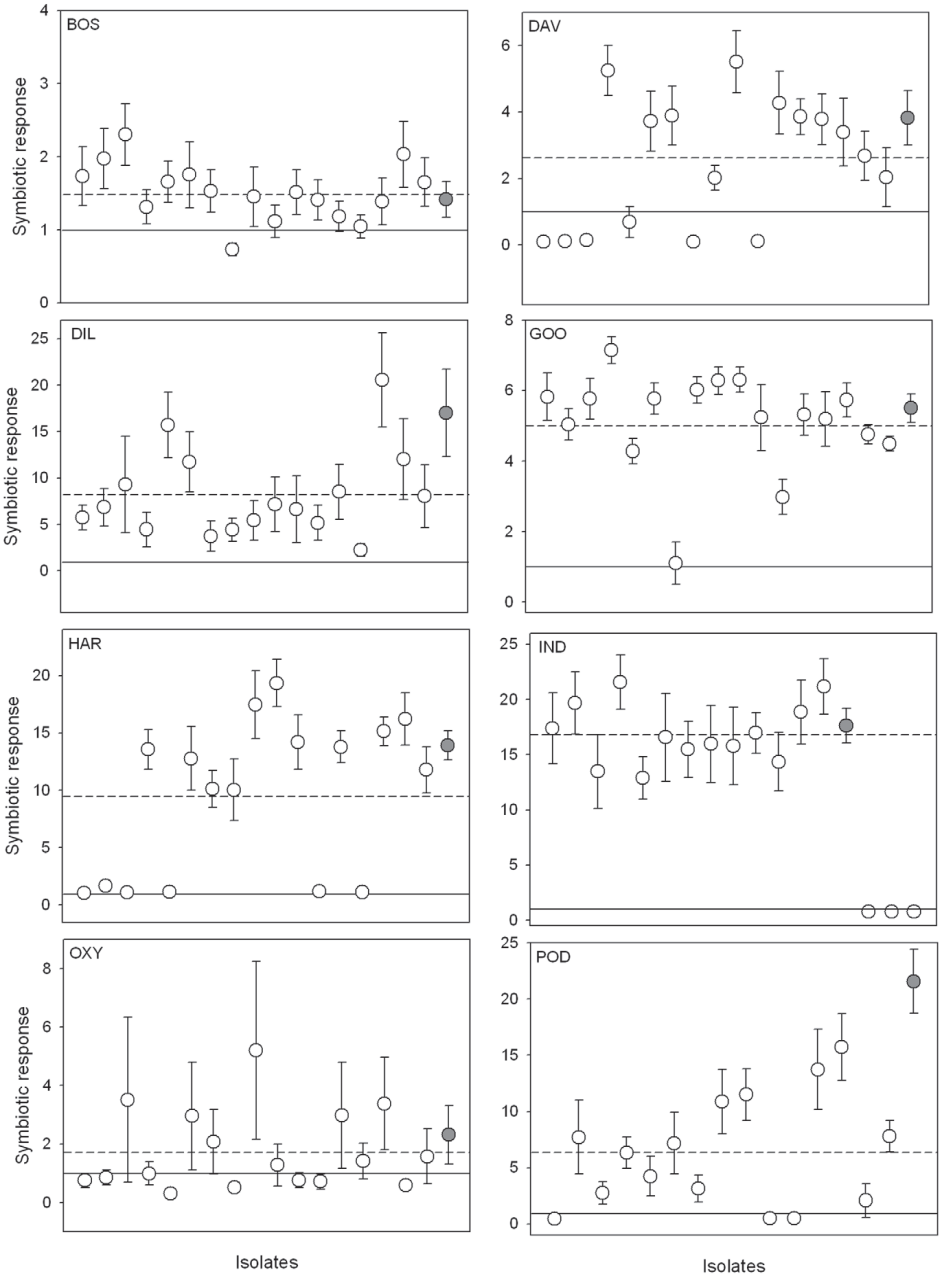
Symbiotic response of the eight host plants to rhizobial strains originally collected from the same host species, and to the broadly effective *Acacia* control strain (WG; filled circles). The solid line at 1 on the y-axis indicates the growth response level where the host did not gain or lose anything from the rhizobial interaction relative to the N^−^ control treatment. Symbiotic response values <1 indicate a negative response, and values >1 indicate a positive effect of inoculation. The dashed line is the average symbiotic response to all rhizobial strains. Error bars are based on standard errors of means (if not visible, they are smaller than the symbols). BOS = *Bossiaea foliosa*, DAV = *Daviesia ulicifolia*, DIL = *Dillwynia retorta*, GOO = *Goodia lotifolia*, HAR = *Hardenbergia violacea*, IND = *Indigofera australis*, OXY = *Oxylobium ellipticum*, and POD = *Podolobium ilicifolium*. Note the different y-axis scales between figure panels.

There was considerable variation in host species responses, both in terms of nodulation as well as in growth responses (*P*<0.0001 for both; Table 2 and Fig. 1). For example, all but one plant (out of 130) of *I. australis* had nodules, while 70% of *O. ellipticum* plants were un-nodulated. For all the other genera, there were at least some host×strain inoculation combinations that did not result in nodules being formed (overall average nodulation across hosts = 75%; *P*<0.0001; Table 2). The broadly effective acacia strain (WG) nodulated 100% of individual plants for most genera; a notable exception was *O. ellipticum* where nodulation with this strain was only successful for 55% of the plants inoculated. This was consistent with the generally poor nodulation observed across the set of strains isolated from that host.

When the outcome of the interaction was measured as host dry weight, host species differed significantly in their overall level of symbiotic response (*P*<0.0001; Table 2). Some host species clearly gained little additional benefit from inoculation (e.g. *B. foliosa*; Fig. 1) while others demonstrated substantial increases in dry weight with rhizobial partners relative to the uninoculated controls (e.g. *I. australis*; Fig. 1). In addition to the overall differences between host species in their response to inoculation *per se*, there was also significant variation among individual rhizobial strains with regard to the benefits conferred on their host plants (*P*<0.0001; Table 2 and Fig. 1), although the degree of variation in performance among strains differed substantially for different host species. With regard to control strain WG, nodule formation did not differ between this strain and the others (*P* = 0.9985). However, plant growth performance was consistently as good as or better with this strain than the average performance of sympatric strains (Fig. 1). This difference was not statistically significant across all host species (*P* = 0.2025), but contrast tests for individual hosts revealed that for *P. ulicifolium*, although WG only nodulated 56% of the plants, it was significantly more effective than the sympatric strains (*P* = 0.0165).

**Figure 2:**
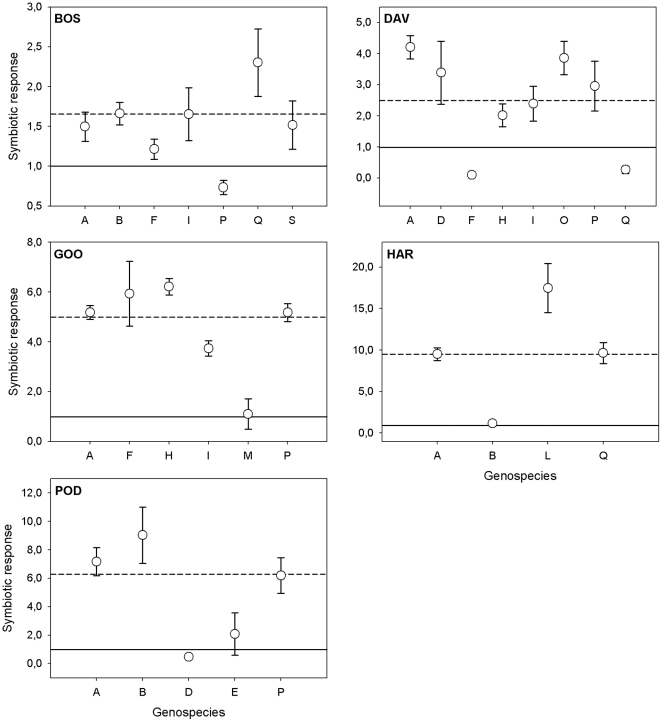
Symbiotic response of five genera of host plants to rhizobial strains classified as members of distinct phylotypes based on RFLP analysis (note that strains belonging to a given phylotype potentially represented isolates from more than one host). The solid line at 1 on the y-axis indicates the level where the host did not gain or lose anything from the rhizobial interaction relative to the N^−^ control treatment. The dashed line is the average symbiotic response to all rhizobial phylotypes. Error bars are based on standard errors of means (if not visible, they are smaller than the symbols). Host species abbreviations are as in Fig. 1. Note the different y-axis scales between figure panels.

**Figure 3:**
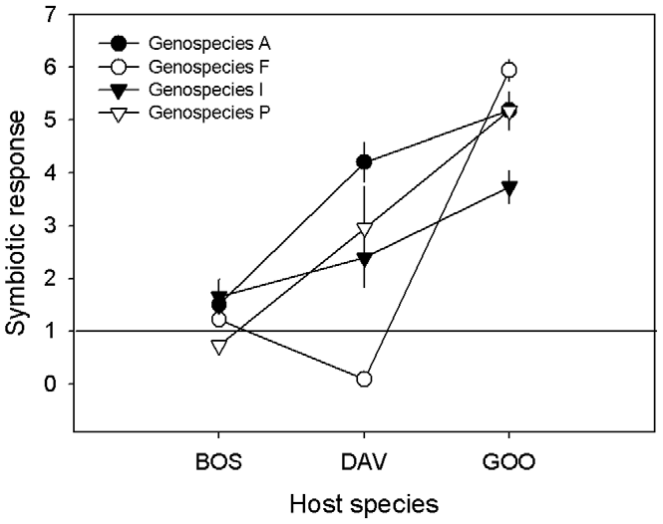
The symbiotic response of four genera of host plants to a common subset of rhizobial phylotypes (i.e. those where each host was represented by one or more of its own strains). The solid line at 1 on the y-axis indicates the level where the host did not gain or lose anything from the rhizobial interaction relative to the N^−^ control treatment. Error bars are based on standard errors of means (if not visible, they are smaller than the symbols). Host species abbreviations are as in Fig. 1.

**Figure 4:**
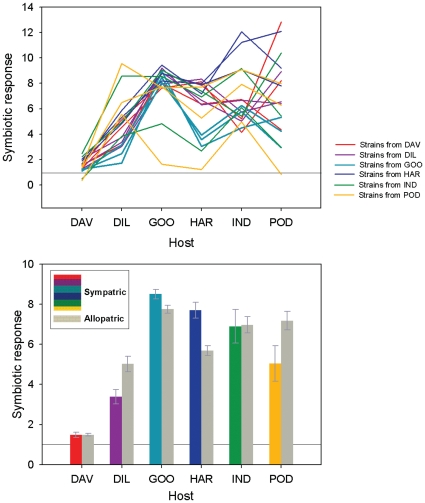
Host growth responses to sympatric and allopatric rhizobial strains. (a) The symbiotic response of six host plants to rhizobial strains originally collected from a given host species and tested with all hosts. The three strains collected from the same host are depicted with the same colour and the colours correspond to those in Fig. 4b. (b) The symbiotic response of hosts to the same rhizobial strains grouped as sympatric (originally collected from the same host species), and allopatric (strains originally collected from other hosts species). Error bars are based on standard errors of means. In both (a) and (b) the solid line at 1 on the y-axis indicates the level where the host did not gain or lose anything from the rhizobial interaction relative to the N^−^ control treatment. Host species abbreviations are as in Fig. 1.

When rhizobial strains were classified into phylotypes based on RFLP banding patterns, the groups did not differ in whether they nodulated their host or not (average nodulation across phylotypes = 85%; Table 2). However, there were clear differences among phylotypes with regard to the growth responses elicited in their host plants (*P* = 0.0051; Table 2 and Fig. 2). Several rhizobial phylotypes were represented among the isolates originating from three of the host species (*B. foliosa*, *D. ulicifolia* and *G. lotifolia*). For this subset of the data, we were able to evaluate the extent to which phylotype performance varied across hosts. The results showed that nodule formation was only affected by the host species (*P* = 0.0207; Table 2). However, symbiotic response (host growth) varied both among the three host species and among the four phylotypes, and there were also strong phylotype×host species interactions (*P* = 0.0078; Table 2 and Fig. 3). For example, rhizobial phylotype F had a negative effect on the growth response of *D. ulicifolia* (relative to the uninoculated N^−^ control), while the highest positive growth response was measured in the interactions between phylotype F and *G. lotifolia* (Fig. 3). This host could be characterised as a generalist as it responded well to inoculation with most rhizobial strains, both its own (Fig. 1) and those of other host species (see below and Fig. 4).

### Experiment II: among-host species inoculations

As expected, there was a consistently high level of nodulation in the second experiment (average percentage of plants nodulated in Experiment I by the subset of symbiotically effective strains used for Experiment II was >95%). Host species differed significantly in their symbiotic response to these strains, while strains did not consistently elicit high or low growth responses in the hosts (*P*<0.0001 and *P* = 0.5373, respectively; Fig. 4a). However, the interaction between host species and strain origin was highly significant (*P*<0.0001; Fig. 4a). In the second analysis where strains were classified as sympatric or allopatric, host species did not have consistently higher or lower growth responses when they were inoculated with sympatric strains (i.e. ones originally sampled from that species) as compared to allopatric strains (those originating from other host species).

Instead, host plants differed significantly in how they responded to strains of sympatric and allopatric origin (host species×sympatry/allopatry interaction: *P*<0.0001; Table 3 and Fig. 4b). Thus, the symbiotic response of *D. ulicifolia* and *I. australis* did not differ with sympatric strains compared to strains originating from other host species, although clearly *D. ulicifolia* was overall far less responsive to inoculation *per se*. In contrast, two of the host species (*G. lotifolia*, *H. violacea*) demonstrated significantly higher growth with their sympatric strains while two host species (*D. retorta*, *P. ilicifolium*) achieved the highest growth with strains originating from other host species (Fig. 4b). Nodulation was overall 5% higher for allopatric interactions compared to sympatric host-strain interactions, and this difference was statistically significant (88% vs. 93%, respectively; *P* = 0.0297; Table 3).

**Table 3:** Analyses of variation in nodulation and growth performance from the across-host inoculation trial (Experiment II) where each legume species was inoculated with its own three most effective sympatric rhizobial strains as well as the three most effective allopatric strains from each of the other host species.

Source	Estimate for random effects (± S.E.)	*Z/F*	*P* value
**Nodule formation**			
Strain (Rhizobial origin)	2.22±1.08	2.06	0.0197
Residual	0.58±0.03	22.78	<0.0001
Host plant_5, 1038_		17.63	<0.0001
Rhizobial origin_5, 12_		0.59	0.7045
Sympatry-Allopatry_1, 1038_		4.74	0.0293
**Symbiotic response**			
Strain (Rhizobial origin)	0.19±0.08	2.34	0.0096
Residual	0.52±0.02	22.70	<0.0001
Host plant_5, 1016_		80.11	<0.0001
Rhizobial origin_5, 12_		1.00	0.4599
Sympatry-allopatry_1, 1031_		0.01	0.9236
Host plant×sympatry-allopatry_5, 1031_		5.58	<0.0001

*Z* statistics are given for random effects and *F* statistics for fixed effects.

## Discussion

Native legumes and their associated soil symbionts (rhizobial bacteria) are of ecological importance in many plant communities, and knowledge of the evolutionary history and distribution of these associations has advanced considerably in recent decades [40], [51]–[53]. Much of the more detailed work on symbiotic effectiveness and rhizobial diversity has focused on variation within host taxa [12], [54], [55], or among host taxa within a genus [16], [56]. However, little is known regarding how associations might vary at higher taxonomic levels. Here we evaluate differences in the provision of mutualistic benefits across naturally co-occurring host genera, using comprehensive inoculation experiments. A key finding was that strain origin with respect to these hosts (sympatry-allopatry) was not a consistent predictor of symbiotic response. The direction and magnitude in the response to sympatric and allopatric strains varied significantly among host species indicating that host community structure is likely to play an important role in the maintenance of symbiont variation. These results thus have relevance for theory focused on the persistence of cheaters in mutualistic associations [21], [57], [58].

### Variation in symbiotic effectiveness within host species

As in previous studies of associations between native legumes and rhizobia [12], [16], we found considerable variation in symbiotic effectiveness (i.e. host growth promotion) among strains associated with a particular host species. Not only were there differences in effectiveness among rhizobial strains, there was significant variation among hosts in their growth responses to inoculation with their own strains. For example, both *Goodia lotifolia* and *Indigofera australis* nodulated effectively, and grew well, with all sympatric strains (Fig. 1). These two species generally also nodulated and grew well with allopatric strains derived from the other host genera, indicating that these hosts can be considered as generalists. Some species, such as *Daviesia ulicifolia* and *Hardenbergia violacea*, exhibited considerable heterogeneity in their growth responses to their own strains; some strain combinations were highly effective, while a significant proportion were clearly worse than the N^−^ control (Fig. 1). Finally, for hosts such as *Oxylobium ellipticum*, few if any strain combinations were effective (Fig. 1), despite these strains having been originally isolated from that host.

Of particular interest was the finding of significant variation among rhizobial phylotypes in symbiotic performance within a given host (over and above variation among individual strains). Not only do these results indicate that rhizobial phylotypes have relevance in an ecological context (i.e. predicting host growth responses), but they contribute to the ongoing debate about the functional relevance of bacterial species in the wider microbial literature [32]–[34]. Our study provides additional data that further support results from a recent comprehensive study [35]. In that study, molecular analyses of rhizobial community structure from 60 sites across southeastern Australia, together with extensive inoculation trials using two *Acacia* spp. present at those sites, found that site-level differences in rhizobial genetic diversity could explain a significant proportion of the variance in growth performance observed in the glasshouse. The mechanisms underpinning such differences among phylotypes are unclear. However, our results suggest that contrary to expectations based on the extensive horizontal transfer of genes associated with symbiosis [51], [59]–[61], evolutionary history and genetic background are likely to be important determinants of ecological performance in symbiotic bacteria.

### Among-species interactions

When we cross-inoculated a subset of host species with their own and each others most effective strains, we found that host species responded in qualitatively different ways to sympatric and allopatric rhizobial strains (Fig. 4). This ranged from species that clearly preferred their own strains (*G. lotifolia*, *H. violacea*), to species that responded well to both sympatric and allopatric strains (*Daviesia ulicifolia*, *I. australis*), and those that, somewhat surprisingly, performed significantly better with allopatric strains (*P. ilicifolium*, *Dillwynia retorta*). This diversity of responses was observed despite the fact that the rhizobial strains selected for the cross-species inoculation experiment were the most effective symbionts on their own hosts (as determined from the nodulation and growth data from Experiment I). Moreover, the majority of the strains used in Experiment II were grouped within a single phylotype (although clearly there can be genotypic variation within a phylotype). Interestingly, strains from both indiscriminate hosts (*D. ulicifolia*, *I. australis*) as well as those preferring sympatric strains (*G. lotifolia*, *H. violacea*) were primarily sampled from the same sites where these hosts co-occurred (Table 1). Thus, not only is there considerable variability in species responses, but clearly the outcome of these interactions can be difficult to predict from knowledge of within-species patterns of symbiotic effectiveness.

Clearly, as shown by the highly significant main effect of host (Table 3) some hosts responded much more to inoculation overall than others (e.g. compare *G. lotifolia*, *D. ulicifolia*; Fig. 4b). Not only did hosts differ in their overall level of responsiveness, but among-strain differences in symbiotic effectiveness varied considerably between hosts. Both *G. lotifolia* and *D. ulicifolia* showed little among-strain variation, while the responses of *P. ilicifolium* spanned the entire observed range (Fig. 4b). Our analyses did not find an overall strain effect, indicating that symbiotic performance of strains also varied across hosts. We note that the highly significant host×strain interaction was observed despite the fact that Experiment II was conducted with a deliberately selected set of symbiotically effective rhizobial strains, representing a small number of phylotypes. Nevertheless, there were a few strains that, across different hosts, either consistently provided clear symbiotic benefits or were ineffective. The maintenance of different rhizobial strategies (mutualism vs. parasitism) is likely to be at least partly a consequence of evolutionary trade-offs in life-history components associated with symbiosis, among-strain competition and reproductive success [62], as has been shown for mycorrhizal fungi [22].

### Concluding Remarks

Considerable effort has focused on advancing a conceptual framework for understanding the factors that determine the magnitude and direction of ecological and coevolutionary trajectories in host-symbiont interactions that fall along the parasitism-mutualism continuum [63]. A recent review predicted that symbiotic associations should become less beneficial with increasing environmental quality and that the association of productivity with symbiont specificity depends on tradeoffs between host range and other life-history parameters [9]. At the same time, biotic complexity is expected to favour generalist pathogens but more specific mutualists. Our results demonstrate significant within and among-species variation in symbiotic effectiveness, ranging from essentially parasitic to highly beneficial associations, but also provide empirical support for the role of host community structure in shaping these interactions. Similarly, negative feedbacks in plant performance caused by specificity in mycorrhizal associations have been implicated as an important determinant of coexistence [24].

Theoretical and empirical studies on the maintenance of variation in host-symbiont associations have largely focused on main-effect differences (i.e. cheaters vs. beneficial mutualists) as might be mediated by trade-offs between the provision of mutualistic benefits and competitive ability among symbionts, or host sanctions [57], [58]. However, we suggest that a broader perspective requires evaluating such effects in concert with a consideration of other factors that are also likely to influence variability within host×symbiont interactions (e.g. local host diversity, physical environment). For example, the generic composition and diversity of rhizobial communities will partly depend on factors such as soil pH [64], salinity [14] or nitrogen levels [65]. Continuing efforts to elucidate the systematics of legumes known to form rhizobial associations [52] will provide tools for exploring community phylogenetic patterns in host-symbiont associations and how these relate to environmental hetergeneity. Combining such approaches will not only enhance basic understanding of symbiotic interactions, but will ultimately result in a greater ability to predict how manipulation of soil biota will contribute to desired ecological outcomes [45], [66].
